# Molecular Characteristics of Epidemiologically Successful *Salmonella* Enteritidis in Poland

**DOI:** 10.1155/tbed/5598487

**Published:** 2025-09-16

**Authors:** Ewelina Kamińska, Magdalena Zając, Magdalena Skarżyńska, Anna Lalak, Katarzyna Bielińska, Pernille Gymoese, Dariusz Wasyl

**Affiliations:** ^1^Department of Omic Analyses, National Veterinary Research Institute (PIWet-PIB), Puławy, Poland; ^2^Department of Microbiology, National Veterinary Research Institute (PIWet-PIB), Puławy, Poland; ^3^Department of Bacteria, Parasites and Fungi, Statens Serum Institut, Copenhagen, Denmark

**Keywords:** antimicrobial resistance, cgMLST, *Salmonella* Enteritidis, ST11, virulence, WGS

## Abstract

Since 2014, the long-term decreasing trend in human salmonellosis, caused mainly by the consumption of *Salmonella*-contaminated poultry products, has stagnated in the European Union (EU). As Poland has been the leading poultry meat producer in the EU since 2014, we analysed whole genome sequences of 275 *Salmonella* (*S*.) Enteritidis strains from the poultry food production chain (*n* = 216) and humans (*n* = 59) (2008–2019) to shed light on the genetic content and relatedness of the *S*. Enteritidis population in Poland. Most (99.3%) of the strains belonged to ST11. Overall, 5.8% of strains possessed at least one antimicrobial resistance gene (ARG), the most common being *qnrB19* (*n* = 9). Mutations in quinolone resistance-determining regions (QRDRs) were observed in 46.9% of strains, and the most common mutation was *gyrA* (S83Y; *n* = 95). In 95.6% of strains, at least one plasmid replicon was detected, with the highest prevalence of IncFII(S)_1 (*n* = 263) and IncFIB(S)_1 (*n* = 262). The composition of *Salmonella* pathogenicity islands (SPIs) was uniform among 96.7% of strains carrying CS54, SPI-1-SPI-3, SPI-5, SPI-6, SPI-9, SPI-12 and SPI-14. Core genome multilocus sequence typing (cgMLST) analysis revealed no apparent clustering based on source or year of isolation. None of the genetic determinants studied here seemed to trigger changes in *Salmonella* epidemiology. However, other factors, such as improvements in reporting and control, could influence infection trends and are, therefore, worth further elucidation.

**Summary**

• Poultry and poultry products seemed to play an important role in the epidemiology of *S*. Enteritidis in Poland.
• 99.3% of *S*. Enteritidis belonged to sequence type ST11.
• 130 virulence factors were detected in the studied collection, and particular strains possessed from 119 to 130 of these genes.
• 5.8% of strains possessed at least one antimicrobial resistance gene (ARG; *bla*_TEM−1_, *bla*_TEM−13*5*_, *sul2*, *tet*(A) and *qnrB19*).
• 95.6% of strains harboured at least one plasmid replicon, with the most common being IncFII(S)_1 and IncFIB(S)_1.

## 1. Introduction

Among over 2600 serotypes of *Salmonella*, many can induce disease both in humans and animals. *Salmonella* (*S*.) Enteritidis is one of the most often transmitted *Salmonella* from animals to humans, causing self-limiting gastroenteritis (diarrhoeal non-typhoidal salmonellosis [dNTS]) or invasive non-typhoidal *Salmonella* disease (iNTS) [1, 2]. Some serovars are more invasive than others, for example, *S*. Enteritidis, which has spread in many countries in a pandemic-like manner [3]. The range of pathogenicity and the dissemination capability of *S*. Enteritidis are connected to various virulence factors promoting host cell invasion, intracellular survival and colonisation [2, 3]. Virulence genes may be present on the chromosome, plasmids, integrated bacteriophage DNA, *Salmonella* pathogenicity islands (SPIs), and *Salmonella* genomic islands (SGIs) [2, 4]. Some of these genomic elements are shared among *Salmonella* species, like SPI-1 (encoding factors crucial for cell adhesion) and SPI-2 (encoding factors important for intracellular survival and replication). However, other factors, like the *Salmonella* virulence plasmid (pSV), being an important virulence factor itself and carrying many different genes, are restricted to only some *Salmonella* subspecies *enterica* serovars, including *S*. Enteritidis [5, 6]. This virulence plasmid is important for *S*. Enteritidis to infect poultry, and its absence can affect this process [5]. Besides virulence factors, plasmids can carry various antimicrobial resistance genes (ARGs). The level of antimicrobial resistance (AMR) in *S*. Enteritidis is relatively low compared to other important serovars like *S*. Typhimurium or *S*. Newport. However, multidrug-resistant (MDR) isolates of *S*. Enteritidis occur worldwide, and their prevalence varies by region, for example, 3.2% in Europe and 40%–81% in China [7].

*Salmonella* strains can be characterised and differentiated below the serovar level using multilocus sequence typing (MLST), which allows for the determination of the genetic relationship between strains of the same serovar [8]. Currently, *S*. Enteritidis sequence type (ST)11 is the most disseminated ST worldwide, according to the Enterobase database, where ST11 comprises 93.9% of all *S*. Enteritidis entries (*n* = 97,848) [9]. Repeated occurrences of multi-country outbreaks linked to ST11 and poultry products have been reported in Europe [10, 11]. All these findings show that poultry constitutes a recurrent risk for salmonellosis worldwide, thus highlighting the need to reduce the prevalence of *Salmonella* in the food production chain, including chicken products [8]. Furthermore, the epidemic success of *S*. Enteritidis ST11, among other STs and, the reasons for this domination are worth elucidating.

In 2019, *S*. Enteritidis was associated with 86.1% of confirmed salmonellosis cases in Poland, while the European Union/European Economic Area (EU/EEA) average was 50.3% [12, 13]. *S*. Enteritidis constituted 50% of all *Salmonella* serovars isolated from eggs, 24.8% from layer flocks, and 25.2% from broiler meat in the EU. Furthermore, over 50% of these *S*. Enteritidis isolates were reported in Poland [13]. Since 2014, an increase in salmonellosis cases in humans has been observed in Poland. In 2015, the percentage of *S*. Enteritidis as a causing factor exceeded 80% again, and this rate is maintained currently [12, 14–21]. In contrast, the EU has seen a stagnation in salmonellosis cases since 2012, although with an increased proportion of *S*. Enteritidis cases. In 2023, the total number of cases in the EU was 77,486, and *S*. Enteritidis was involved in 70.8%. The overall tendency for *Salmonella* infections did not change significantly between 2019 and 2023 [22]. In Poland, the number of cases in 2023 was 10,348, which constituted an increase in comparison to 2022 (6575), and *S*. Enteritidis was the most often isolated serovar (83.8%) [21]. These findings support the suspicion that the control programmes intended to scale down the prevalence of *Salmonella* along the food chain have not been effective [13, 23]. The One Health European Joint Programme Project called ADONIS (Assessing determinants of the non-decreasing incidence of *Salmonella*) was launched in 2020 to assess reasons for the stagnation/reversal tendency in *S*. Enteritidis in humans and poultry in the EU. To investigate this, a cross-sectorial approach was applied, and different factors at the levels of primary production, epidemiology/exposure and the bacterium itself were taken into account [24]. In 2014, Poland rose as a major poultry meat producer in the EU (13.9% of the total). Over half (1.6 million tonnes) of its production is exported, mainly to the intra-EU market [25]. Considering this, the presented study has focused the analyses on *S*. Enteritidis isolates in Poland from poultry and humans, their genomic characteristics, and their genetic relationship. We aimed to identify or propose possible genomic traits involved in its successful sustainability in poultry production and spread to humans.

## 2. Materials and Methods

### 2.1. Data Collection

This study analysed 275 sequences of *S*. Enteritidis (Table 1). The 216 sequences, originating from *S*. Enteritidis strains isolated between 2008 and 2019 from primary poultry production and poultry food products, were selected from the collection of the National Reference Laboratory for Salmonellosis located at the National Veterinary Research Institute (PIWet-PIB) in line with the ADONIS selection criteria (metadata available in Supporting Information_1). Isolates of different origins and from each source and year (preferably different province, or different county, or at least different identification numbers of places of origin) and with the complete metadata were selected to meet the required number of strains per isolation source set for Poland. Due to an insufficient number of isolates, some years are over-represented. Additionally, 59 sequences (fasta format) originating from human salmonellosis cases in Poland (metadata and accession numbers are available in Supporting Information _2_SE_human_PL) were arbitrarily selected and downloaded from the Enterobase database (accessed June 26, 2023) with the following sequence selection criteria applied: Collection year <2020; Country: Poland; Source Type: Human; Strain according to SeqSero2: *Salmonella* Enteritidis.

### 2.2. Culturing, Whole-Genome Sequencing, and Assembly of *Salmonella* Enteritidis

Deep-frozen strains of *S*. Enteritidis were retrieved on xylose lysine deoxycholate (XLD) and nutrient agar, and cultured under standard conditions (overnight incubation at 37 ± 1°C). Pure cultures were used for DNA isolation by the Maxwell Rapid Sample Concentrator (RSC48) using the Cultured Cells DNA Kit (Promega). DNA libraries were prepared using the Kapa HyperPlus Kit or the Nextera XT Kit. Whole genome sequencing was performed on MiSeq (V3; 2 × 300 bp; Illumina). Adapter trimming and quality control of raw reads were done using Trimmomatic v0.36 [26] and FastQC v0.11.5 [27], respectively. Corrected reads were merged by bbmerge [28]. The draft genomes were assembled using paired and merged reads using SPAdes v3.9.0 with the '--careful' option [29]. The genome quality statistics of all sequences were determined using the quality assessment tool for genome assemblies (QUAST) v4.5 [30].

### 2.3. Bioinformatic Analysis

The *Salmonella In Silico* Typing Resource (SISTR) v1.1.1 was used for serotype determination [31]. STs were detected using mlst v2.22.1 [32] and the PubMLST database [33]. SPIs, ARGs, plasmids replicons and virulence genes were detected using Abricate v1.0.0 [34] with the databases SPIFinder (version 2020-12-04) [35], NCBI (version 2023-07-18) [36], PlasmidFinder (version 2023-03-17) [37] and virulence factor database (VFDB) (version 2023-07-28) [38], respectively. The mutations in quinolone resistance-determining regions (QRDRs) were detected using Staramr v0.10.0 [39] with the PointFinder database (version 2021-02-01). Applied thresholds for the minimum nucleotide identity (ID) and gene coverage (*Q*) were as follows: AMR and virulence genes with 95% ID and 95% *Q*, plasmid replicons with 95% ID and 90% *Q*, and SPIs with 90% ID and 60% *Q*. Core genome MLST (cgMLST) was analysed using chewBBACA v2.1.0 [40] with the Enterobase scheme [41]. Minimum spanning trees (MSTs) were calculated using the GrapeTree and MSTreeV2 algorithms [42]. Visualisations of the MST were made in iTOL [43] and GrapeTree [42]. Identification of genetic clusters was performed using ReporTree v2.3.1 [44] with the clustering method MST (MSTreeV2, GrapeTree) [42].

### 2.4. Statistical Analysis

The statistical significance of differences in the prevalence of mutations in QRDR and the *shdA* gene was determined using the chi-square test. The Mann–Whitney–Wilcoxon Test was used to determine statistically significant relatedness between plasmid presence and the number of virulence genes. The alpha level of 0.05 was applied as a minimum level of statistical significance. All statistical analyses were done in RStudio (2022.7.2.576) [45].

## 3. Results

### 3.1. Serovar Prediction and MLST Typing

All strains were confirmed as *Salmonella enterica* subsp. *enterica* serovar Enteritidis with the antigenic formula 1,9,[12]:g,m:- according to the White-Kauffmann-Le Minor scheme [46]. The majority of isolates belonged to sequence type ST11 (*n* = 273), with single cases of ST8211 and ST8212.

### 3.2. Comparative Analysis

The range of allelic differences between strains was 0–332 among ST11 and up to 889 when taking into account all STs. Eleven groups of strains with no allelic differences were observed, containing from two to five strains. The biggest group comprised strains isolated between 2013 and 2015 from broilers (200806_3037, 200730_3010), broiler meat (200806_3039), laying hens (200730_3019) and breeding flocks (200724_2977). In two cases, no allelic differences were noted in strains from the food production chain and humans, for example, from broilers in 2019 (200703_2882) and from humans in 2019 (SAL_TC6245, SAL_TC6631).

**Figure 1:**
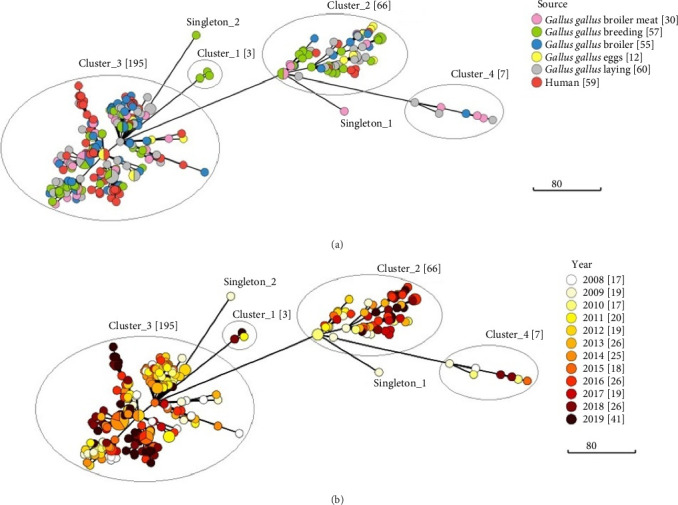
Minimum spanning trees of 273 S. Enteritidis strains (ST11) with cluster definition at 69 allelic differences showing source (a) and year (b) distribution.

Regions of cluster stability were detected at four levels: 42, 69, 116 and 149 allelic differences. Clustering at the level of 69 allelic differences was arbitrarily chosen for presentation, as this clustering was most congruent with the metadata (Figure 1). Strains of ST11 were grouped into four clusters and two singletons. The largest cluster, cluster 3 (*n* = 195), contained the majority (51/59) of all human-origin strains, constituting 26.2% of all strains in this cluster. In comparison, cluster 2 (*n* = 66) contained 12.1% (*n* = 8) human strains. No isolation source patterns were identified. Both clusters comprised strains isolated throughout the studied term (2008–2019) in slightly different proportions. In cluster 2, a predominance of strains isolated after 2014 was observed (59.1%), while in cluster 3, strains isolated between 2008 and 2014 were more common (55.9%). The analysis of the timespan distribution shown in the MST can be disturbed due to the disproportionately large percentage of human strains isolated in 2019 (24/59) and an unequal number of representatives for individual years.

When clustered at the threshold of 5 allelic differences, 30 clusters of varying sizes could be distinguished (Supporting Information_3_MST). The largest cluster, 28, was composed of 58 strains from all sources and all years, except 2008 and 2010. The first occurrence within this cluster was in 2009 (broiler flocks), and it could be observed until 2019, when 23 strains were isolated: 18 from humans, three from broiler flocks, one from laying flocks and one from breeding flocks.

Two strains other than ST11 were not shown in the MST (Figure 1). According to the distance matrix, the most distinct strain was ST8212 (minimum allelic differences—787) and would be clustered as an additional singleton in the MST. Strain ST8211 showed a more similar cgMLST profile, with a minimum allelic difference of 20 and would be included in cluster 4.

### 3.3. AMR

**Figure 2:**
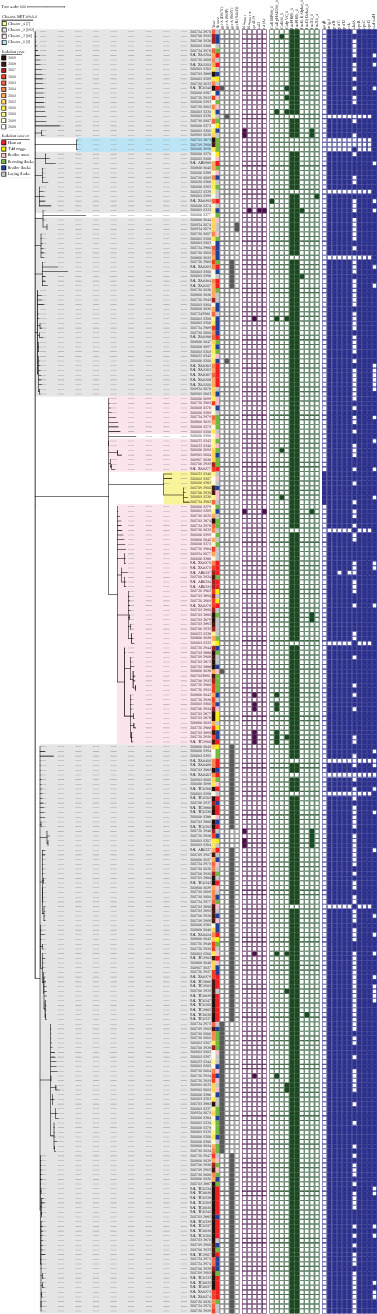
A minimum spanning tree of 273 *Salmonella* Enteritidis (*ST11*): clusters (tree colours); isolation year (colour strip); isolation source (colour strip), QRDR mutations (grey): *gyrA* (*D87Y*), *gyrA* (*S83F*), *gyrA* (*S83Y*), *gyrB* (*S464Y*); antimicrobial resistance genes (purple): *bla*_*TEM−1*_, *bla*_*TEM−135*_, *qnrB19*, *sul2*, *tet*(*A*); plasmid replicons (green): Col(MP18)_1, Col(pHAD28)_1, Col156_1, ColpVC_1, IncFIB(S)_1, IncFII(S)_1, IncI1-I(Alpha)_1, IncI2(Delta)_1, IncX1_1, IncX1_4; virulence genes with abundance level 0.36%–95.6% (blue): *gogB*, *mig-5*, *pefB*, *pefC*, *pefD*, *rck*, *shdA*, *spvB*, *spvC*, *spvD sseI/srfH*.

Just 5.8% (*n* = 16) of *S*. Enteritidis strains possessed at least one ARG. ARGs occurred in 6.9% (*n* = 15) of poultry food production chain strains (mostly broilers, *n* = 9) and 1.7% (*n* = 1) of human origin (Figure 2). The following AMR mechanisms were identified: beta-lactamases (*bla*_TEM−1_, *n* = 6; *bla*_TEM−135_, *n* = 1), class A tetracycline efflux pumps (*tet*(*A*), *n* = 2) as well as resistance genes to fluoroquinolones (*qnrB19*, *n* = 9) and sulfamethoxazole (*sul2*, *n* = 1). The most commonly observed ARG—*qnrB19*, was identified in strains isolated from broilers (10.9%), broiler meat (7.1%) and humans (1.8%).

In addition, nearly half (129/275) of strains possessed mutations in QRDRs (Figure 2). Point mutations were more prevalent in human-originating strains (69.5%) than in strains isolated from food production (40.7%) (*p*  < 0.001). The most common mutations were: *gyrA* (S83Y) (34.5%) and *gyrA* (D87Y) (10.9%), as well as a few cases of *gyrA* (S83F) (0.7%), *gyrB* (S464Y) (0.7%). Point mutation *gyrA* S83Y was more often observed in strains from humans (67.8%) than in food chain strains (25.5%) (*p*  < 0.001). The opposite observation concerned the D87Y *gyrA* mutation (1.7% and 13.4% of strains, respectively) (*p*  < 0.05). Almost all QRDR-mutant strains were included in cluster 4, with a single exception in cluster 3 (Figure 2).

One strain (0.4%), originating from broiler meat from 2010, showed multidrug resistance (MDR—resistance to at least three classes of antimicrobials) genotype, carrying resistance genes to ampicillin (*bla*_TEM−135_), sulfamethoxazole (*sul2*) and tetracycline (*tet*(A)), but without QRDR mutations.

### 3.4. Plasmid Replicons and Their Association With AMR

**Figure 3:**
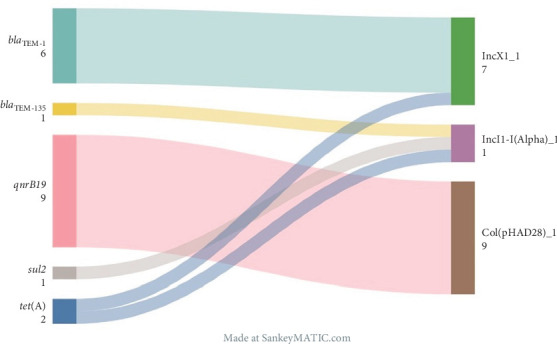
Detected antimicrobial genes and their association with plasmid replicons. The numbers under the names of features indicate the prevalence.

In 95.6% of strains, at least one plasmid replicon was detected, including ST8211 and ST8212 (Figure 2). Of the 10 replicons identified, the most common were IncFII(S)_1 (*n* = 263) and IncFIB(S)_1 (*n* = 262), followed by the less frequent Col(pHAD28)_1 (*n* = 10), IncX1_1 (*n* = 9), ColpVC_1 (*n* = 9), Col156_1 (*n* = 3), IncI1-I(Alpha)_1 (*n* = 2) and single cases of Col(MP18)_1, IncI2(Delta)_1 and IncX1_4. All detected ARGs were localised on the same contigs as plasmid replicons (Figure 3).

The ST8212 strain had a unique plasmid replicon profile possessing only IncFII(S)_1, while ST8211 was similar to most of ST11, having IncFIB(S)_1 and IncFII(S)_1 replicons.

### 3.5. SPIs

In the studied collection, 10 types of SPIs were detected (Supporting Information_1). The vast majority of strains (96.7%) were uniformly carrying SPI-1, SPI-2, SPI-3, SPI-6, SPI-9, SPI-12, SPI-14 and CS54. This included all ST11 and ST8211 strains originating from the poultry food production chain.

### 3.6. Virulence-Associated Genes

Genomic analysis revealed 130 virulence genes (Supporting Information_1). Most identified virulence factors (*n* = 98) were present in all strains, for example, *sopBDE2* and *invABCFGHIJ*, which are important for host cell invasion. There were also other virulence genes (*n* = 30) with slightly lower (range 84.4% to 99.6%) occurrence, for example fimbrial genes *pefBCD* encoding adhesins. Three genes were significantly less frequent: *sinH* (*0.4%*), *gogB* (*2.9%*) and *shdA* (*38.2%*) (Figure 2), and exhibited source patterns. The *shdA* gene contributing to efficient and prolonged faecal shedding was four-fold more often (*p*  < 0.001) seen in strains of the poultry food production chain (*n* = 100; 45.7%) than in strains from humans (*n* = 6, 10.2%). The anti-inflammatory gene *gogB* was found only in animal-originating strains (*n* = 8), which clustered together on the minimum spanning tree (Figure 2). The *sinH* gene occurred once in the ST8212 strain.

Strains with at least one plasmid replicon harboured statistically significantly (*p*  < 0.001) more virulence genes (average = 128.11; SD = 1.02) than those without replicons (average = 120.46; SD = 1.27). Strains with no plasmid replicons lacked the following genes: *mig-5*, *pefBCD*, *rck* and *spvBCD* (Figure 2). Plasmid-borne virulence genes *spvB*, *spvC* and *spvD* were identified in 263 of the 275 strains examined in this study and co-occurred with the IncFII(s)_1 replicon. Many other virulence genes were located within SPIs (CS54_island, SPI-1, SPI-2 and SPI-3) (Supporting Information_1).

## 4. Discussion

The ADONIS project intended a Europe-wide analysis of *S*. Enteritidis of food chain and human origin. Since the latter component was not represented from Poland, the current work intended to shed light on *S*. Enteritidis strains at the national level and analyse genetic factors that could influence the above-mentioned phenomenon in the epidemiology of *S*. Enteritidis.

### 4.1. Genetic Relatedness Between Human and Poultry Food Production Chain Isolates

Notably, epidemiological links between human and poultry isolates can be inferred. More than half (*n* = 34) of the isolates clustered with strains from the poultry food production chain, with five or fewer allelic differences, which is an often applied threshold for defining a cluster in outbreak analysis [10, 47, 48]. This suggests these strains may be genetically related, especially when considering the timespan of isolation. Furthermore, three human isolates exhibited identical cgMLST profiles to strains of food chain origin. Taking into account the above findings and the fact that no source or year distribution pattern was observed, we could suspect that strains were circulating in the primary production of poultry, occurring in different sources along the food production chain over time and, consequently, presented one of the sources of salmonellosis in humans. Poultry and poultry products seemed to be an important factor in the epidemiology of *S*. Enteritidis in Poland, as well as in the EU, as it was stated in annual EU Zoonoses reports [11, 13, 22, 23]. Similar findings were concluded in the studies performed in Singapore and Lebanon [8, 49]. Strains studied in this paper are only a small subset of all *S*. Enteritidis present in the PIWet-PIB collection. Still, within this limited sample, we have already spotted significant relationships.

Another important observation was the domination of ST11 in the food production chain and human strains. Again, similar *S*. Enteritidis ST11 domination in both poultry and humans was observed in Colombia and Lebanon [49, 50]. In Australia (Queensland and New South), although ST11 prevailed, it ranged between 64.5–78.9% among clinical isolates. In both studies, distinct clusters were observed, within which the percentage of STs was different, suggesting the *S*. Enteritidis population in Australia was more diverse in terms of STs [51, 52]. Additionally, in Australia, the main serovar causing salmonellosis was *S*. Typhimurium, while *S*. Enteritidis was mainly associated with travellers [52]. In Asian regions, *S*. Enteritidis epidemiology differs. ST1925 dominated in Malaysia (63.7%) and Singapore (70.3%), surpassing the second most prevalent ST11 (26.5% and 9.7%, respectively) [8, 53]. This is congruent with Enterobase, where ST11 accounts for most of the entries both in Europe (93.4%, 58153/62275, including Poland, 97.3%) and North America (95.5%, 22349/23394). In comparison, a lower percentage of ST11 could be observed in Asia (88.0%, 916/1041). The *S*. Enteritidis populations in Europe and North America were less diverse in terms of STs compared to those in Asia and Australia. No entries were found in Enterobase with ST8211 and ST8212, and there were no literature reports on these STs [9]. Although the observation pinpointed that these STs did not play a significant role in the epidemiology of *Salmonella* infections, it confirmed the possibility of the occurrence of a diverse population of the pathogen that led to the huge domination of a single ST for unknown reasons.

### 4.2. Virulence Genes and SPIs

The epidemic success of ST11 may be a result of its pathogenicity traits. Various virulence factors contribute to *Salmonella* pathogenicity. Most virulence genes are clustered in the so-called SPIs on the chromosome [53]. There are five major SPIs: SPI-1, SPI-2, SPI-3, SPI-4 and SPI-5 [54]. SPI-1 and SPI-2 encode type 3 secretion systems (T3SS), SPI-5 carries genes co-regulated with either SPI-1 or SPI-2, and SPI-3 is involved in both gut colonisation and intracellular survival. All five SPIs were present in all strains, which aligns with findings from other studies [7, 55]. However, in the Singapore strains, the prevalence of SPI-1, SPI-3 and SPI-5 was slightly lower (98.9–99.3%) [8]. SPI-4, as in our study (97.5%), was present in most strains (98.9%). Interestingly, SPI-2 was not detected in any of the strains, in contrast to a uniform distribution in the current study [8]. Within SPI-1 and SPI-2, the virulence genes *avrA*, *sprB*, *sicAP* and *sipABCD*, which encode T3SS, were detected. These two islands are the most significant for the pathogenicity of *S*. Enteritidis and play a crucial role in the colonisation of systemic sites in chickens [54]. The *invA* gene associated with SPI-1 was present in all isolates. Similar findings were found in other studies [55], while some authors reported a slightly lower frequency [56]. SPI-3, required for survival in macrophages and growth in low-magnesium conditions, was associated with the following genes: *mgtC*, *mgtB* and *misL*. Localisation of these genes on SPI-3 was previously documented [54, 57, 58].

The virulence genes *csgA*, *csgD* and *sopB/sigD* involved in biofilm formation were detected in all isolates, as also documented in other studies in Poland and Colombia [59, 60]. Adhesion-related genes were rarely observed, that is, s*hdA* (38.5%) and *sinH* (0.34%) as well as a phage-encoded type III-secreted substrate *gogB* (2.9%). In China, the prevalence of these genes among *S*. Enteritidis isolated from animals and humans was higher: 99.6%, 100.0% and 16.1%, respectively [61].

Two strains, ST8211 and ST8212, had similar virulence gene profiles to ST11. One gene, *sinH*, was uniquely present in the ST8212 strain. This gene, associated with the adhesion and invasion of host cells, was present in all clinical isolates of different serovars, including *S*. Enteritidis associated with iNTS in Israel [62]. Although ST8212 carried the *sinH* gene observed in strains causing iNTS, the presence of *sinH* did not translate into a broader epidemiological importance of ST8212. From the above, we might conclude that the epidemic success of a certain ST or strain is more complex than the presence of one virulence factor.

Virulence genes are also often present on mobile genetic elements. We noticed that strains possessing plasmids had statistically significantly higher numbers of virulence genes than strains with no plasmids (*p*  < 0.001). Virulence factors that were associated with plasmid replicon were: *mig-5*, *pefBCD*, *rck* and *spvBCD*, similar to other studies [63, 64]. Plasmid-borne virulence genes such as *spvBCD* were identified in 95.6% of strains examined in this study and associated with IncFII(S)_1 replicon, thus confirming results that most of the strains carried mobile genetic elements. The *Spv* locus is one of the most important virulence determinants facilitating growth and proliferation in a host organism [7, 65]. According to other studies, the *spv* operon is present in varying frequencies ranging from 15.1% to 94.9% [7]. Our results showed a slightly higher frequency of these genes in human (96.6%) and poultry food production chain (95.4%) strains.

### 4.3. Plasmids

Plasmids are essential vectors of resistance and virulence factors. The plasmid genes can be transmitted via horizontal transfer, thus contributing to the dissemination of crucial traits for epidemic success [66]. The plasmid replicons IncFII(S)_1 and IncFIB(S)_1 were the most frequently detected. This is, however, slightly incongruent with a multicounty collection study showing just a 50% occurrence of IncFII(S) and IncFIB(S) replicons [35]. As in our study, the results of the Lebanese research showed that most strains carried 2–3 replicons [49]. The occurrence of plasmid replicons did not exhibit any (evident) pattern in the MST. It seems these genetic elements did not, at first glance, contribute to the dissemination of certain strains (success of particular clusters), and therefore, did not influence the epidemiological trends of *S*. Enteritidis infections.

Plasmids can play a role in AMR transmission, including in *S*. Enteritidis, which could be supported by the localisation of all detected ARGs on the same contigs as plasmid replicons. In one case, the IncI1-I(Alpha)_1 replicon was associated with the *bla*_TEM−135_, *tet*(A), and *sul2* genes and resulted in an MDR genetic profile of the strain. These types of plasmids have previously been reported to carry ARGs in other *Salmonella* serovars, for example, Infantis, as well as in other species like *E. coli* isolated from broiler and pig farms in Belgium [67, 68]. The co-occurrence of Col(pHAD28)_1 replicon and the *qnrB19* gene observed in our study was also detected in *S*. Infantis from the Netherlands and Brazil [68] and isolates of *S*. Hadar in Germany [69].

### 4.4. AMR in *S*. Enteritidis Isolates

There are different determinants of acquired AMR, including mutations or ARGs. In the studied collection, all ARGs were suspected to be plasmid-mediated, and the most often observed ARG was *qnrB19* (3.3%). The presence of plasmid-mediated quinolone resistance (PMQR) in *Salmonella* spp. originating from Europe, the United States and Brazil was previously documented in other studies [69, 70]. In Poland, the *qnrB19* gene was detected in 17% of *Salmonella* spp. clinical isolates and was more often observed than other variants of PMQR (*qnrB36*, *qnrB82*, *qnrB67*) [71]. In our study, no other variants were detected. In the USA, *qnrB19* prevalence among swine sources of *Salmonella* spp. was detected at 80.0% [70]. Similarly, a study conducted in Brazil showed that *qnrB19* was the most frequent PMQR and was found in 74.4% of *Salmonella* spp. isolated from the poultry and swine production chains [72]. Studies from around the world, including those from Poland, showed a higher prevalence of *qnrB19* in *Salmonella* spp [69–72]. and *S*. Enteritidis alone [71] than we detected. It could be influenced by the source of isolation, as well as country-dependent factors and antimicrobial use (fluoroquinolones).

Other ARGs were even less prevalent, which is congruent with European trends. The resistance to ampicillin (*bla*_TEM_ genes), sulfamethoxazole (*sul2*) and tetracycline (*tet*(A)) was noted at 2.2%, 0.4% and 0.4%, respectively. According to an EFSA/ECDC report in 2022, resistance to these compounds in *S*. Enteritidis was low, ranging from 5.1% for ampicillin, 4.3% for tetracycline and 3.1% for sulfamethoxazole [73]. Results showed that such resistance was rarely observed in Europe in *S*. Enteritidis, although these antimicrobials are widely used in veterinary medicine to treat infections in animal husbandry.

Among the studied collection, 5.8% of strains possessed at least one ARG. In comparison, among *S*. Enteritidis in Australia, the prevalence of ARGs varied according to clades (0–69.6%), with an average of 44.2% [49]. In an earlier study, 18% of strains with ARGs were reported [52].

Another genetic factor underlying fluoroquinolone resistance is QRDR mutations. Point mutations in *gyrA* or *gyrB* were detected in 46.9% of the *S*. Enteritidis strains. In another study conducted among clinical *Salmonella* isolates in Poland, 60.0% of the isolates possessed a mutation in *gyrA* or *parC*, with *gyrA* S83Y (45.0%) and D87Y (21.0%) as the most prevalent mutations [71]. In Brazil, from *Salmonella* spp. isolated from the poultry and swine production chains, 30.2% of isolates had a single mutation only in *gyrA* [72]. In Singapore, mutations in QRDRs (*gyrA*, *gyrB*, *parC* and *parE*) were observed among 33.7% of *S*. Enteritidis isolates. The most prevalent were mutations in *gyrA* D87Y (15.9%), D87G (5.4%) and S83Y (4.7%) [8]. Our findings are congruent with those of other studies, showing a higher prevalence of *gyrA* to other QRDR mutations. The percentage of QRDR mutations is slightly lower in studies from different countries than in Poland.

Exclusively, one isolate presented an MDR genetic profile (0.4%) that is lower than European and USA averages (3.2% and 2.2%, respectively), and disproportionately lower than in China (41.0–80.0%) or sub-Saharan Africa (42.0%) [7]. In other studies conducted in Poland, the MDR percentage among *S*. Enteritidis isolated from various foods (e.g., eggs, confectionery) was 1.9% [74].

Overall, the detected resistance in *S*. Enteritidis was relatively low, so it could not be the primary reason for the epidemiological success of this serovar, and other factors should be elucidated.

## 5. Conclusion

The studied collection presented highly homogeneous characteristics in terms of ST (99.3% of ST11) and a considerable level of homogeneity in terms of SPIs (96.7% shared the same SPI pattern). Although some variability was noted in plasmid replicons and plasmid-associated genes, no clustering based on these factors or any common source or year of isolation was identified. AMR of *S*. Enteritidis was relatively low, so it could not play a major role in the epidemiology of this serovar, and other factors should be considered. Based on these findings, we were unable to identify any single and specific determinant that could contribute to changes in *Salmonella* epidemiology in Poland. However, other factors such as improvements in reporting, control and surveillance programmes of *Salmonella* cases in humans or the premature loosening of *Salmonella* control programmes at the primary production chain could influence infection trends.

Moreover, the epidemic success of ST11 in Poland, as well as in other parts of the world, remains unclear. The ST8211 strain showed high similarity to ST11 in terms of the genetic content analysed in this study, suggesting similar virulence and dissemination capabilities. However, there were no reports on this ST in the Enterobase or literature. This strain was isolated in 2008 from breeding flocks, and the reason for its unsuccessful persistence is puzzling, as are the overall features and epidemiology of most, if not all, members of the *Salmonella* genus.

## Figures and Tables

**Table 1:** Sequences of *S*. Enteritidis by source and year of isolation.

Isolation source	2008	2009	2010	2011	2012	2013	2014	2015	2016	2017	2018	2019	Total
*Gallus gallus* laying flocks	5	5	5	5	5	5	5	5	5	5	5	5	60
*Gallus gallus* breeding flocks	5	5	4	5	5	5	5	5	5	5	5	5	59
*Gallus gallus* broiler flocks	4	5	4	5	4	5	4	4	5	5	5	5	55
*Gallus gallus* broiler meat	3	3	3	3	3	3	3	1	3	1	4	0	30
Table eggs	1	1	1	2	2	0	0	0	3	0	0	2	12
Human	0	0	0	0	0	8	8	3	6	3	7	24	59
Total	18	19	17	20	19	26	25	18	27	19	26	41	275

## Data Availability

The data that support the findings of this study are openly available in the European Nucleotide Archive at https://www.ebi.ac.uk/ena/browser/home.

## Supporting Information

Supporting Information 1 A table containing the list of all strains with metadata (year and source of isolation), genomic characterisation results (ST, presence of antimicrobial resistance and virulence genes, plasmids, SPIs, mutations in QRDRs) and clustering results at different thresholds, and accession numbers.

Supporting Information 2 A table containing complete metadata and accession numbers of human-origin strains.

Supporting Information 3 A JSON file containing the minimum spanning tree of 273 *Salmonella* Enteritidis (ST11) with complete metadata uploaded (source and year of isolation, results of ARGs, virulence genes, plasmid replicons, SPIs, mutations in QRDR searching and clustering results at different thresholds). The JSON file can be uploaded here: https://achtman-lab.github.io/GrapeTree/MSTree_holder.html and manipulated according to user needs.
